# My Team of Care Study: A Pilot Randomized Controlled Trial of a Web-Based Communication Tool for Collaborative Care in Patients With Advanced Cancer

**DOI:** 10.2196/jmir.7421

**Published:** 2017-07-18

**Authors:** Teja Voruganti, Eva Grunfeld, Trevor Jamieson, Allison M Kurahashi, Bhadra Lokuge, Monika K Krzyzanowska, Muhammad Mamdani, Rahim Moineddin, Amna Husain

**Affiliations:** ^1^ Institute of Health Policy, Management and Evaluation University of Toronto Toronto, ON Canada; ^2^ Department of Family and Community Medicine University of Toronto Toronto, ON Canada; ^3^ Division of General Internal Medicine St. Michael's Hospital Toronto, ON Canada; ^4^ Institute for Health System Solutions and Virtual Care (WIHV) Women's College Hospital Toronto, ON Canada; ^5^ Department of Medicine University of Toronto Toronto, ON Canada; ^6^ Temmy Latner Centre for Palliative Care Mount Sinai Hospital Toronto, ON Canada; ^7^ Department of Medical Oncology and Hematology Princess Margaret Cancer Centre University Health Network Toronto, ON Canada; ^8^ Li Ka Shing Centre for Healthcare Analytics Research and Training St. Michael's Hospital Toronto, ON Canada; ^9^ Leslie Dan Faculty of Pharmacy University of Toronto Toronto, ON Canada; ^10^ Institute of Clinical Evaluative Sciences Toronto, ON Canada

**Keywords:** MeSH: Internet, professional-patient relations, interdisciplinary communication, neoplasms, adult, chronic disease, continuity of patient care, patient care team, communication, outcome assessment (health care)

## Abstract

**Background:**

The management of patients with complex care needs requires the expertise of health care providers from multiple settings and specialties. As such, there is a need for cross-setting, cross-disciplinary solutions that address deficits in communication and continuity of care. We have developed a Web-based tool for clinical collaboration, called Loop, which assembles the patient and care team in a virtual space for the purpose of facilitating communication around care management.

**Objective:**

The objectives of this pilot study were to evaluate the feasibility of integrating a tool like Loop into current care practices and to capture preliminary measures of the effect of Loop on continuity of care, quality of care, symptom distress, and health care utilization.

**Methods:**

We conducted an open-label pilot cluster randomized controlled trial allocating patients with advanced cancer (defined as stage III or IV disease) with ≥3 months prognosis, their participating health care team and caregivers to receive either the Loop intervention or usual care. Outcome data were collected from patients on a monthly basis for 3 months. Trial feasibility was measured with rate of uptake, as well as recruitment and system usage. The Picker Continuity of Care subscale, Palliative care Outcomes Scale, Edmonton Symptom Assessment Scale, and Ambulatory and Home Care Record were patient self-reported measures of continuity of care, quality of care, symptom distress, and health services utilization, respectively. We conducted a content analysis of messages posted on Loop to understand how the system was used.

**Results:**

Nineteen physicians (oncologists or palliative care physicians) were randomized to the intervention or control arms. One hundred twenty-seven of their patients with advanced cancer were approached and 48 patients enrolled. Of 24 patients in the intervention arm, 20 (83.3%) registered onto Loop. In the intervention and control arms, 12 and 11 patients completed three months of follow-up, respectively. A mean of 1.2 (range: 0 to 4) additional healthcare providers with an average total of 3 healthcare providers participated per team. An unadjusted between-arm increase of +11.4 was observed on the Picker scale in favor of the intervention arm. Other measures showed negligible changes. Loop was primarily used for medical care management, symptom reporting, and appointment coordination.

**Conclusions:**

The results of this study show that implementation of Loop was feasible. It provides useful information for planning future studies further examining effectiveness and team collaboration. Numerically higher scores were observed for the Loop arm relative to the control arm with respect to continuity of care. Future work is required to understand the incentives and barriers to participation so that the implementation of tools like Loop can be optimized.

**Trial Registration:**

ClinicalTrials.gov NCT02372994; https://clinicaltrials.gov/ct2/show/NCT02372994 (Archived by WebCite at http://www.webcitation.org/6r00L4Skb).

## Introduction

With advances in medical care enabling people to live longer, patients with chronic diseases and their families have increasingly complex care needs requiring the expertise of many health care providers from multiple settings and more frequent use of the health care system [1-3]. Important contextual information is not consistently exchanged between health care providers, and coordinated delivery of patient care as a team is lacking [4-7]. As such, there is a need for solutions that are cross-organizational, cross-setting, and that improve continuity of care, defined as the extent to which delivery of care by different providers is coherent, connected, and timely [8].

Organizations such as the Institute of Medicine and the Agency for Healthcare Research and Quality have called for solutions that build on the growing momentum of health information technology to address the deficits in continuity of care and coordinated delivery of care [9-13]. With over 80% of the populations of Canada and the United States having access to the Internet and mobile phones [14], Web and mobile-based communication are ideally positioned to improve the sharing of knowledge, expertise, and decision making between providers (ie, collaboration) [15], to involve patients and, by extension, improve continuity of care [16,17]. Solutions have generally been limited to one-to-one secure messaging or email, possibly as additions to information systems such as patient health records [18].

Reviews on the impact of tools for patient-physician communication have shown promising evidence of improvement on such outcomes as patient self-efficacy, satisfaction with care, and on clinical/psychosocial outcomes [19,20]. However, few tools exist with the express intent of facilitating secure *team*-based communication, which can enable sharing of information between different providers, across health events and settings, and promote collaborative care [21]. Previous studies examining tools that enable patients to communicate with their health care team have been observational in design and focused their examination on implementation in the pediatric [22], general primary care [23], elderly [24], and cerebral palsy [25] populations. These studies did not consider such outcomes as continuity of care, which is particularly pertinent to the patient population with complex care needs.

In this study, we evaluated a Web-based tool for clinical collaboration, called Loop. The purpose of Loop is to assemble care teams that include patients and caregivers in order to facilitate communication and collaboration [26]. We conducted a pilot randomized controlled trial in a population of patients with advanced cancer, as prototypical of a population with complex care needs [27,28]. Our objective was to evaluate the feasibility of integrating a tool like Loop into current care processes and to capture preliminary measures of the effect of Loop on continuity of care, quality of care, symptom distress, and health care utilization.

## Methods

### Trial Design

We conducted a 15-month multicentered, nonblinded, pragmatic pilot cluster-randomized controlled trial (cRCT), called the My Team of Care study, allocating participants to receive access to Loop as the intervention arm or to usual care as the control arm. The unit of randomization was at the level of the physician, and the unit of analysis was at the level of the individual patient.

See Multimedia Appendix 1 for the CONSORT EHEALTH checklist [29].

### Setting and Participants

The study took place at the Temmy Latner Center for Palliative Care at Mount Sinai Hospital and Princess Margaret Cancer Center in Toronto, Ontario, Canada, from January 2015 to April 2016. The Temmy Latner Center is the largest home-based palliative care program in Canada, consisting of 23 palliative care physicians. The Princess Margaret Cancer Center is a University of Toronto‒affiliated research hospital with 46 medical oncologists and 42 radiation oncologists. At both study sites, patients generally access their physician through visits and telephone messages; some health care providers are contactable via email.

Participants consisted of eligible patients plus their principal cancer physician (oncologist or palliative care physician), and if interested, their family caregiver (informal, unpaid). For the intervention arm, additional health care providers as identified by the patient were also invited to participate as members of the circle of care to use Loop.

Eligible patients were aged 18 or older; had stage IV cancer or stage III cancer with poor prognosis as determined by their oncologist (a survival prognosis of ˃3 months but ˂2 years); Eastern Cooperative Oncology Group (ECOG) performance status score of 0, 1, or 2, as assessed by their oncologist or palliative care physician at time of enrollment; English literacy and language competency to provide informed consent and complete questionnaires; and patients or caregivers had access to a computer and Internet. Exclusion criteria were currently receiving or a candidate for hormone therapy for breast or prostate cancer (given the impact on prognosis); impaired mental status assessed with the Bedside Confusion Scale [30] (score of ≥2 suggesting cognitive impairment); or participation in another study precluding participation in this study.

### Intervention

Participants randomly allocated to the intervention arm received access to Loop. Loop is a secure online communication tool for team-based clinical collaboration that enables patients and caregivers to communicate asynchronously with multiple members of the health care team involved in providing their direct care (ie, not individuals hired for the purpose of research), as well as for health care providers including physicians, nurses, and allied health professionals to communicate with each other.

The development of Loop followed a user-centered design approach [31] with substantial end-user and stakeholder involvement (including caregivers, health care providers from several specialties, and patients with different conditions). As described by Kurahashi et al [26], this process included initial needs assessments, ethnographic observational studies, and affinity diagramming leading to the development of a prototype. This was followed by simulation activities, usability testing in laboratory and real-world settings (ie, home, clinics, hospitals, offices), and piloting with patients and clinical teams.

**Figure 1:**
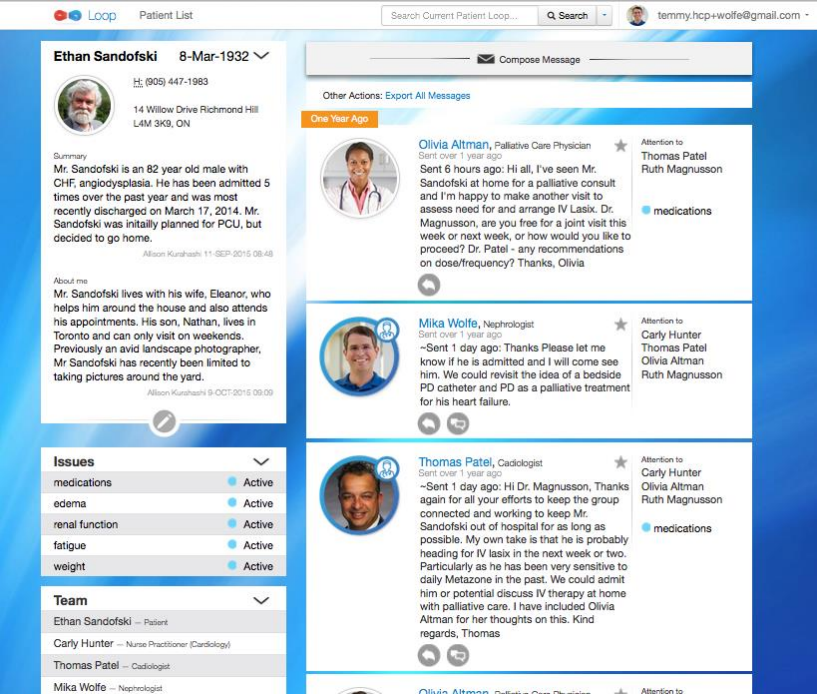
Screenshot of the Loop interface on desktop computer.

**Figure 2:**
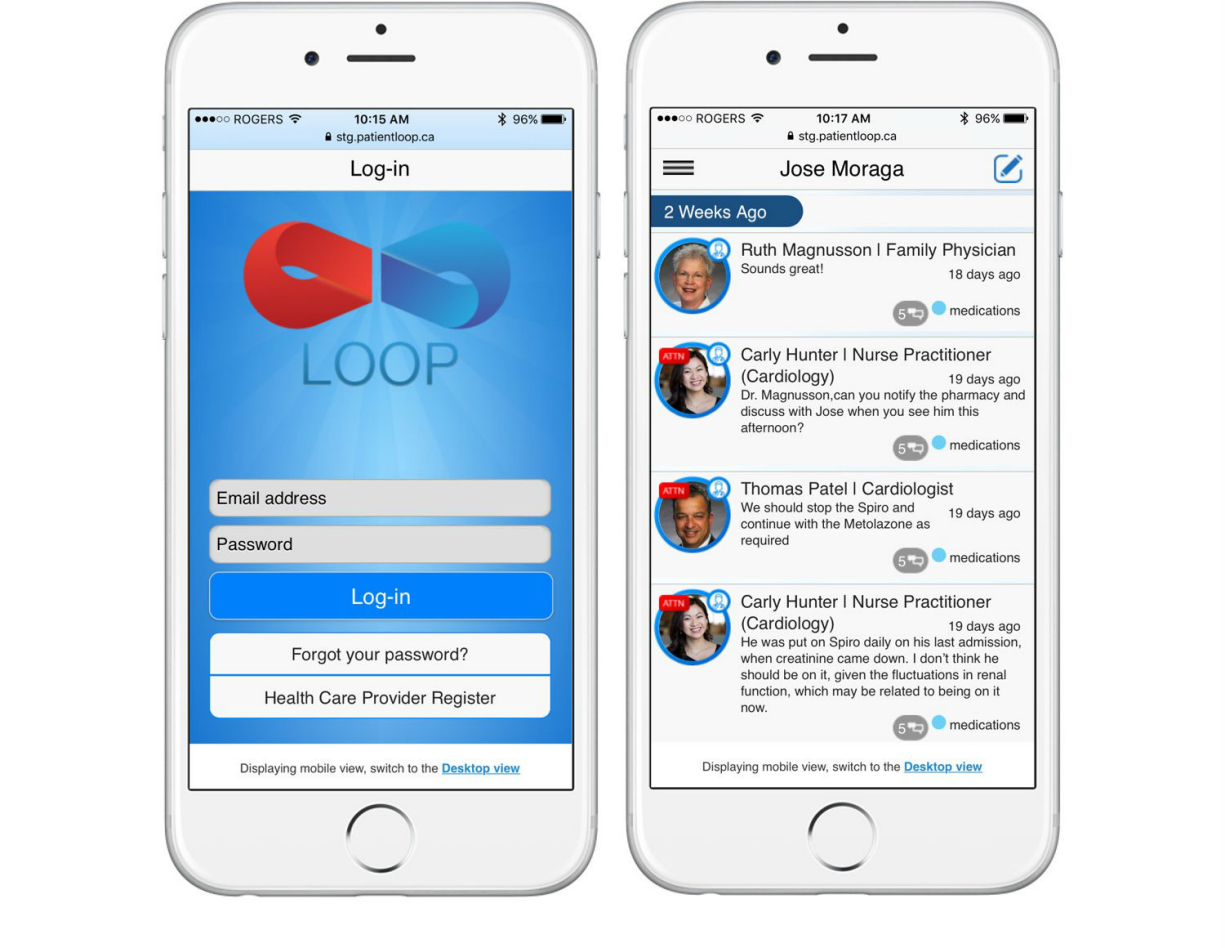
Screenshot of the Loop interface on mobile phone.

Loop was developed with an intuitive interface to allow for use ideally without prior training. A patient profile and space are created that can be viewed by the patient, health care providers, and caregivers on a computer or mobile phone after logging in with an email address and password (Figures 1 and 2). Each patient’s Loop is a secure space partitioned from other Loops and can be joined only if involved in the patient’s care and authenticated by a study administrator. Health care providers may be a part of multiple patients’ Loops, but patients cannot access the Loops of other patients. On the main page, individuals can write and post text-based messages. All messages posted by any member of a patient Loop can be read and responded to by members of that given Loop. All entries remain on the patient space, allowing for previous posts to be viewed. The messages are threaded in conversations and can be searched using various filters. In addition to posting messages, users may label posts with user-defined “tags” and an “Attention To” feature that specifies individuals to be alerted to a post by a generic email. No updates to the system were made during the trial.

### Recruitment and Study Procedures

In order to ensure that there would be at least one health care provider on each team, medical/radiation oncologists or palliative care physicians were recruited first (“initiating physician”), randomized, and patients from their practices were then approached in clinic prior to appointments or over the phone. Physicians at the two study sites were notified of the study through announcements at educational rounds, and physicians who expressed interest in participating were followed up with directly.

All participants provided consent and were asked to complete a baseline demographics form and an internally developed 11-item survey on participants’ access, comfort, and usage of computers and Internet [32]. Initiating physicians and patients in the intervention arm were then invited to register on Loop. Registration involved completion of a form requesting participant name, address, and, in the case of health care providers, professional license number. Once registered on the tool, an intervention patient was considered active in the study. Through the tool, patients could invite family caregivers and additional health care providers to join their Loop. Study administrators contacted, explained the study, obtained consent from all additional members of a patient Loop prior to registration, and posted an introductory message welcoming participants. Study administrators were part of patient Loops only for the purpose of providing assistance during registration and with using the tool, as requested. When a patient was no longer part of the study, Loops remained open for 2 weeks to allow for message exchange records to be exported and saved.

Use of the tool and the type of communication that could occur on the tool were not prescribed. The intervention protocol did not specify intent to replace existing care practices or methods of communication; the intervention was additive. As Loop was not meant to be used for urgent communication, this was reinforced during the consent process and in the Loop terms of use.

Recruitment of initiating physicians and their patients was conducted similarly in the control arm.

### Usage of Loop

Usage of the intervention was evaluated from message exchange transcripts and audit data from Loop. Data included time to registration on Loop from consent date, number of participants who registered on Loop, number of messages exchanged, number of times additional features (Attention To, Tagging) were used, and number of views and posts by participants.

We conducted a content analysis of Loop messages to summarize the content of messages on patient Loops. Two coders independently reviewed messages exchanged in each patient Loop and assigned categories thematically that emerged from the data (see Multimedia Appendix 2 for coding framework and definitions) [33]. Categories were assigned to messages and any responses or follow-up posts. Categories were assigned only once per Loop and not quantified. If multiple categories were perceived in a single post, then each was included once as a category identified in that particular Loop. Messages posted by administrators to welcome team members were excluded.

### Outcomes

The primary feasibility outcomes were participant recruitment rate and implementation fidelity defined as the proportion of participants who were randomized, completed the baseline demographics and computer usage questionnaire, and if in the intervention arm, registered on Loop, with ≥70% completion indicating feasibility success. This threshold was selected as an adequate threshold to justify further study and has been suggested previously in the literature [34].

Secondary outcomes were measured using standardized instruments to assess the impact of Loop. Mean difference over the course of the study (from baseline to months 1, 2, and 3) for each instrument was calculated. Patient-reported continuity and coordination of care was measured with the 8-item Picker Ambulatory Cancer Care Survey (Picker) Continuity and Coordination subscale questionnaire. The Picker scale is scored by summing absolute positive responses, divided by the total number of responses (scores range from 0-100, with higher scores being better), and a minimal clinically important difference of 10 points has been previously found to be significant [35,36]. The Palliative care Outcomes Scale (POS) was used to assess patient-reported quality of care and well-being. The POS is a 12-item self-administered questionnaire (total scores range from 0-40, with higher scores being worse); a difference of one point on each item is considered clinically meaningful [37-39]. The Edmonton Symptom Assessment Scale (ESAS) is a 9-item, patient-reported questionnaire of symptom intensity (pain, tiredness, nausea, depression, anxiety, drowsiness, appetite, well-being, shortness of breath) with each item rated from 0 (worst) to 10 (best). Individual symptoms are summed for the Total Symptom Distress score (ranging from 0-90, with higher scores indicating worse symptom distress) [40]. Health care utilization was measured as number of visits to the emergency department and number of hospitalizations and was self-reported at each monthly assessment using the Ambulatory and Home Care Record [41].

Data were collected monthly for 3 months from baseline (four time points). Questionnaires were distributed electronically using online surveys emailed to study participants via Research Electronic Data Capture version 6.16.7 [42], a data management system hosted at the Applied Health Research Center of St. Michael’s Hospital, Toronto, Ontario. Patients who did not respond within 1 week were followed up via a reminder email or telephone call and were considered lost to follow-up if not reachable after four contacts. We piloted outcome assessments and survey administration prior to the study. We also collected qualitative interview data at assessments, which will be reported separately.

### Randomization and Blinding

This study was designed as a cRCT with initiating physicians recruited first and randomized in order to minimize contamination between study arms. Participating patients were allocated to the study arm to which their initiating physician had been randomized. Randomization was done by a statistical team independent of the study using a computer-generated randomization sequence consisting of permuted blocks of varying size, and assigned initiating physicians in a 1:1 ratio to the intervention and control arms. It was not possible to blind patients completely to study arm, but control patients provided consent without being informed of the existence of another arm. This was done to minimize bias of control patients basing their decision to participate on study arm assignment. Control patients were informed that they were taking part in a study on patient-physician communication to improve health care delivery and care management. Investigators and initiating physicians were aware of study arm assignment.

### Sample Size and Statistical Analysis

A formal sample size calculation was not computed, as this was a pilot study with primary feasibility outcomes. We set a target sample size of 20-25 patients per study arm, which has been previously justified as sufficient for pilot evaluations [34,43]

The primary analysis was intention-to-treat with available cases. We did not make adjustments for missing data but secondarily report comparison of data for complete cases (participants who completed outcome assessments at all time points). Descriptive statistics were used to describe each study arm. Analysis compared mean change scores and unadjusted differences in mean change scores on the preliminary effectiveness outcomes between study arms. Statistical tests of difference were not conducted since the study was not powered to undertake hypothesis testing.

**Table 1:** Baseline patient and family caregiver characteristics by treatment arm.

Characteristics			Intervention arm (n=21)	Control arm (n=21)
**Patients**				
	Age in years, mean (SD)		60 (12.8)	59.5 (13.8)
	Female sex, n (%)		13 (61.9)	16 (76.2)
	**Primary cancer site, n (%)**			
		Breast	1 (4.8)	10 (47.6)
		Colorectal	2 (9.5)	1 (4.8)
		Lung	3 (14.3)	6 (28.6)
		Prostate	2 (9.5)	0
		Ovarian	0	1 (4.8)
		Thyroid	2 (9.5)	0
		Lymphoma	6 (28.6)	0
		Melanoma	0	1 (4.8)
		Brain	1 (4.8)	0
		Other	4 (19.0)	2 (9.5)
	**Annual household income in CDN$, n (%)**			
		$0-$21,999	2 (9.5)	4 (19.1)
		$22,000-$49,999	2 (9.5)	2 (9.5)
		$50,000-$89,999	7 (33.3)	4 (19.1)
		>$90,000	4 (19.1)	5 (20.8)
		Prefer not to disclose	6 (28.6)	6 (28.6)
	**Primary language, n (%)**			
		English	20 (95.2)	20 (95.2)
		Other	1 (4.8)	1 (4.8)
	Age-adjusted Charlson Comorbidity Index, mean (SD)^a^		5.2 (2.5)	5.8 (1.9)
	**Caregiver, n (%)**			
		Yes	4 (19.1)	6 (28.6)
		No	17 (81.0)	15 (71.4)
	**Highest education attained, n (%)**			
		Primary school	‒	‒
		High school	4 (19.1)	6 (28.6)
		College/University	8 (38.1)	8 (38.1)
		Professional/Graduate degree	9 (42.9)	7 (33.3)
	ECOG score, median (interquartile range)^b^		1.5 (1-2)	1 (1-2)
	**Outcome measures (n=39)**			
		POS, mean (SD)^c^	9.3 (6.8)	9.8 (5.4)
		Picker Continuity and Coordination subscale, mean (SD)^d^	47.9 (28.5)	62.5 (25.3)
		ESAS (Total Symptom Distress Score), mean (SD)^e^	21.2 (17.1)	23.4 (12.9)
**Family caregivers of consented patient participants**			**(n=18)**	**(n=8)**
	Age in years, mean (SD)		57 (15.9)	54 (14.6)
	Female sex, n (%)		9 (60.0)	6 (33.3)
	Missing, n (%)		3 (16.7)	‒
	**Relationship to patient, n (%)**			
		Spouse	7 (38.9)	4 (22.2)
		Immediate family	5 (27.8)	8 (44.4)
		Other	3 (16.7)	‒
	Missing data		3 (16.7)	‒

^a^Age-adjusted Charlson Comorbidity Index is a measure of comorbidity based on risk of mortality. The score is weighted by age, increasing for each decade over age 40 [44].

^b^ECOG scale is scored from 1-5 with 1 being well and 4 indicating complete disability. A value of 5 indicates death.

^c^Mean summed scores are presented for POS with a maximum score of 40. Higher scores indicate worse quality of care.

^d^The Picker Continuity and Coordination subscale is a proportion of total number of positive responses to total number of responses. Higher scores indicate the higher perceived continuity of care.

^e^Mean summed scores are presented for the ESAS with a maximum score of 90. Higher scores indicate higher symptom distress.

### Ethics

All participants provided written, informed consent to participate. Research Ethics Boards of the University Health Network, Mount Sinai Hospital, University of Toronto, and the Community Care Access Centers of Toronto, Ontario, approved the study.

## Results

**Figure 3:**
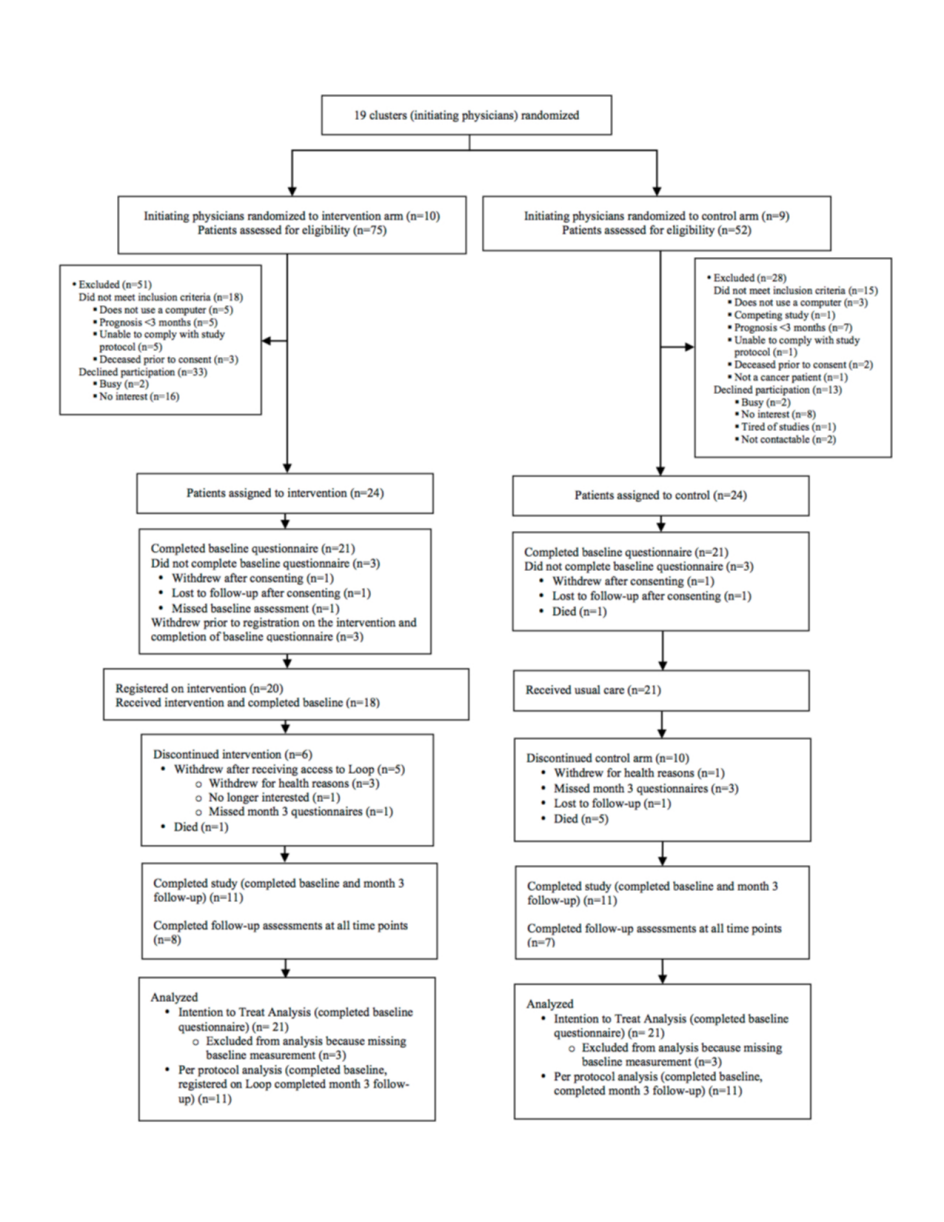
Participant Flow Diagram.

We recruited 10 palliative care physicians and 9 medical oncologists and sequentially randomized 10 to the intervention arm and 9 to the control arm. We assessed 127 patients for eligibility of whom 94 were eligible. We recruited 24 patients each to the intervention and control arms. Figure 3 shows the randomization of clusters (initiating physicians), reasons patients declined to participate in the study, and patient follow-up. In each arm, the baseline questionnaire was completed by 21 patients. In the intervention arm, 18 family caregivers participated and in the control arm, 8 family caregivers participated. There were two instances of initiating physicians from the intervention arm serving as additional health care providers on other intervention patient Loops.

Between arms, there was minimal difference between patients on demographic characteristics, with some modest discrepancies resulting from small sample size (Table 1). There was differential distribution of patients’ primary cancer diagnoses by arm reflecting differences in clinical subspecialty of the participating physicians: lymphoma (6 in intervention vs 0 in control), breast (1 in intervention vs 10 in control) and lung (3 in intervention vs 6 in control). There was minimal difference at baseline in comorbidity and performance status as measured with the ECOG score. Participants were comfortable with using computers and less so Internet-enabled devices (tablets and mobile phones), as described in Multimedia Appendix 2. Initiating physicians in both arms showed similar demographic and practice characteristics (Table 2). All were from academic settings, and most had an alternative payment plan fee structure.

Regarding team assembly in the intervention arm, an average of 3 health care providers, including the initiating health care provider, participated per patient Loop. Patients suggested between 1 to 5 additional health care team members to participate on Loop; 43% (22/51) consented to participate in the study (mean 1.2 per patient [range 0-4]), and 65% (13/20) of patient Loops had an additional health care provider register on the tool (Table 2).

### Usage of Loop

In the intervention arm, 83% (20/24) of patients who consented, registered on Loop (Table 3). In terms of health care provider load, the mean number of patient Loops per initiating physician was 1.6 (range 0-7). Registration on Loop required that the baseline questionnaire be completed beforehand. The time from consent to registration on Loop varied considerably, with the mean time to registration being 39 days. Some patients experienced disease worsening between consent and registration, and one patient delayed taking the step to register for 156 days due to personal circumstances. Over the study period, the majority (85%, 17/20) of Loops had message exchanges, with 45% (13/20) having more than six messages exchanged. During the study, there were 358 logins by all participants: 43 on the mobile version and 315 on the desktop version. Patients viewed their Loops more often relative to their number of posts (a difference of 14.3) compared to initiating physicians viewing and posting to their patients’ Loops (a difference of 3.9).

**Figure 4:**
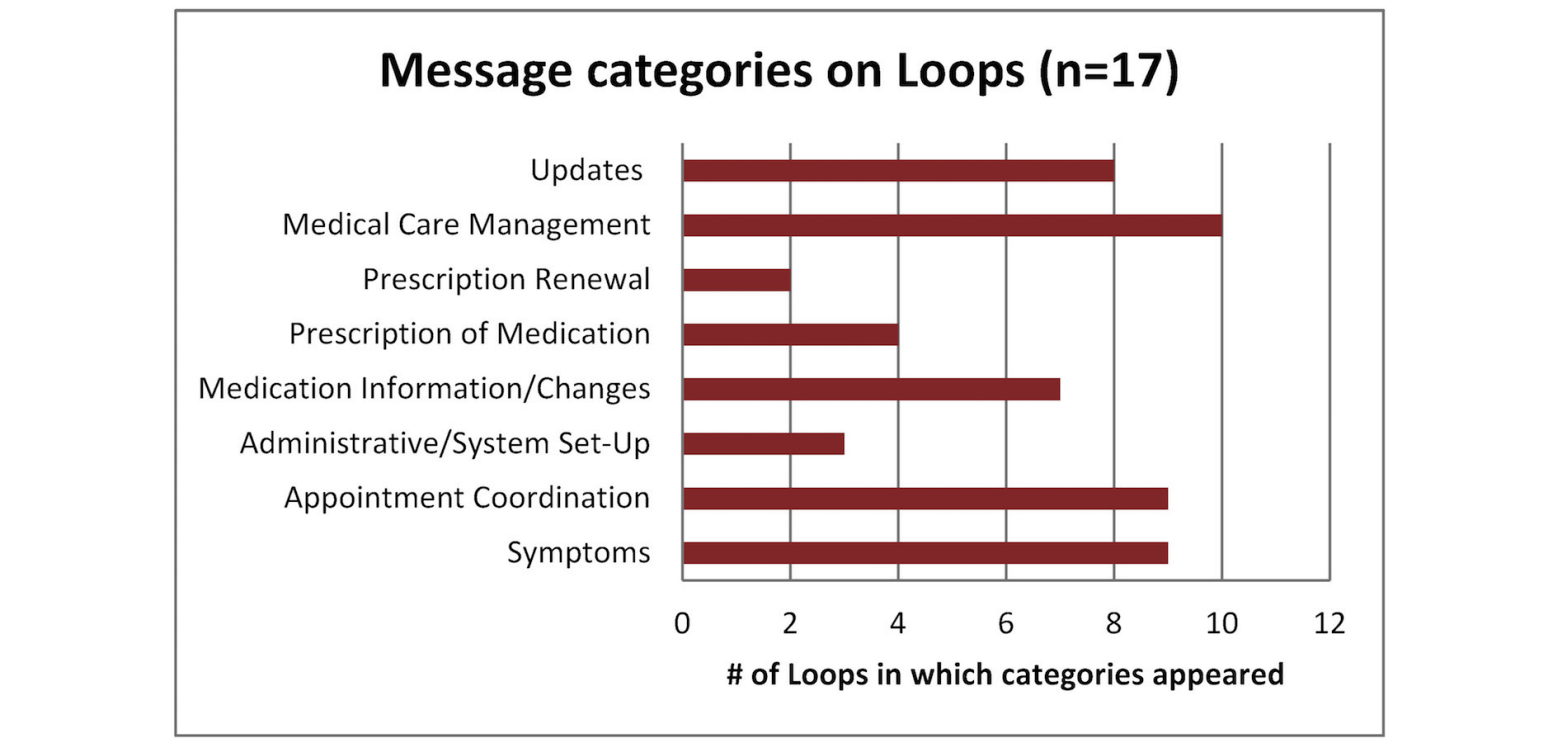
Categories of messages on Loops with messages exchanged.

Content analysis of messages revealed that of Loops with messages exchanged, messages regarding medical care management, reporting of symptoms, and appointment coordination predominated (these categories were identified in 50%, 45%, and 45% of the 20 patient Loops, respectively), while only 10% of the Loops had messages that were prescription-related queries (see Figure 4). No urgent messages were exchanged during the study.

**Table 2:** Baseline health care provider demographics.

Characteristics			Intervention arm (n=10)	Control arm (n=9)
**Initiating physicians**				
	Age in years, mean (SD)		44 (7.9)	43 (6.1)
	Female sex, n (%)		5 (50.0)	3 (33.3)
	Years in health care, mean (SD)		16 (8.8)	15 (6.5)
	**Initiating physician profession, n (%)**			
		Medical oncologist	4 (40.0)	2 (22.2)
		Radiation oncologist	1 (10.0)	2 (22.2)
		Palliative care physician	5 (50.0)	5 (55.6)
	**Primary practice setting, n (%)**			
		Hospital-based	6 (60.0)	4 (44.4)
		Home-based care	4 (40.0)	5 (55.6)
		Other	‒	‒
	**Type of practice, n (%)**			
		Community setting	‒	‒
		Academic setting	10 (100)	9 (100)
	**Practice fee structure, n (%)**			
		Fee-for-service	‒	‒
		Alternate payment plan	8 (80.0)	7 (77.8)
		Salaried	1 (10.0)	1 (11.1)
		Other	1 (10.0)	1 (11.1)
	**Provides after-hours care, n (%)**			
		Telehealth	‒	‒
		Phone support	2 (20.0)	4 (44.4)
		Phone support with visit when needed	6 (60.0)	5 (55.6)
		Other	‒	‒
		None	2 (20.0)	‒
Additional health care providers identified, N			51	
**Additional health care providers^a^** **(n=22)**				
	**Profession, n (%)**			
		Family physician	1 (4.5)	
		Nurse	4 (18.2)	
		Case manager	1 (4.5)	
		Palliative care physician	4 (18.2)	
		Medical oncologist	5 (22.7)	
		Naturopath	1 (4.5)	
		Oncology nurse	1 (4.5)	
		Otolaryngologist	1 (4.5)	
		Personal support worker	1 (4.5)	
		Psychiatrist	1 (4.5)	
		Pharmacist	1 (4.5)	
		Physiotherapist	1 (4.5)	
	Additional health care providers who consented and registered on Loop, n (%)		16 (72.7)	
	Additional health care providers identified per patient, mean (range)		2.4 (1-5)	

^a^Recruited as part of the intervention arm and who provided consent.

**Table 3:** Usage of Loop (intervention arm participants, n=24).

Loop usage			Mean (range) or n (%)
**Loop composition^a^**			
	Patients who registered on Loop (regardless of baseline questionnaire completion), n (%)		20 (83)
	**Initiating physicians (intervention arm, n=10) who, n (%):**		
		Registered on Loop	9 (90)
		Used the tool (posted at least 1 message or viewed a patient Loop)	7 (70)
	Health care providers (including initiating physician) per patient Loop, mean (range)		3 (0-5)
	Additional health care providers suggested by each patient, mean (range)		2.4 (1-5)
	Additional health care providers per patient Loop, mean (range)		1.25 (0-4)
	Patient Loops health care provider is a part of, mean (range)		1.6 (0-7)
	Family caregivers per patient Loop, mean (range)		0.5 (0-1)
**Frequency of use of the tool, n**			Loops, n
	**Messages exchanged per Loop by registered participants (n=20)**		
		0	3
		1-2	5
		3-5	3
		6-10	6
		>10	3
	**Views of a patient’s own Loop by the patient or caregiver (n=20)**		
		0	0
		1-2	3
		3-5	4
		6-10	5
		>10	8
	**Posts to a patient’s own Loop by the patient or caregiver (n=20)**		
		0	6
		1-2	5
		3-5	3
		6-10	4
		>10	2
	**Views of a patient Loop by an initiating physician (n=9)**		
		0	2
		1-2	1
		3-5	2
		6-10	4
		>10	0
	**Posts to all their patient Loops by an initiating physician (n=9)**		
		0	3
		1-2	5
		3-5	1
		6-10	0
		>10	0
**Use of additional features**			
	Time from consent to registration on Loop (days), mean (range)		39 (2-156)
	Times an issue was tagged, mean (range)		1 (1)
	Times Attention To feature was used by a patient or caregiver, mean (range)		3 (0-14)
	Times Attention To feature was used by a health care provider per Loop, mean (range)		0.6 (0-3)

^a^A “Loop” is an aggregation of a patient and/or caregiver and at least the initiating physician allocated to the intervention arm, and registered on the intervention tool.

### Outcomes

For the primary outcomes, the mean number of patients recruited per initiating physician was similar between study arms (in intervention arm 2.4 [range 0-7] in intervention vs control arm 2.7 [range 0-7]) (Table 4). With respect to implementation fidelity, 88% (21/24) of control patients who consented completed the baseline questionnaire and 75% (18/24) of intervention patients who consented completed the baseline questionnaire, along with registering on the tool. Regarding patient retention, in the intervention arm, 3 patients withdrew due to declining health, 1 patient withdrew because they were no longer interested, and 1 patient died. In the control arm, 1 patient withdrew due to declining health, and 5 patients died. Instrument and item completion were approximately proportional to patient retention. Of patients completing the baseline outcome assessment, 52% (11/21) in the intervention arm and 52% (11/21) in the control arm completed Month 3 outcome assessments.

Results described are based on available cases. Mean change scores and unadjusted difference in change scores between study arms for preliminary effectiveness outcomes are presented in Table 5. At Month 3, there was an increase in Picker scale scores (in intervention arm +10.2 [SD 31.5] vs control arm -1.1 [SD 30.3]), a negligible change in POS (in intervention arm +0.8 [SD 4.4] vs control arm +0.5 [SD 5.4]), and an increase in ESAS Total Symptom Distress score (in intervention arm +2.3 [SD 10.7] vs control arm +3.4 [SD 8.7]). The number of patients with emergency room visits self-reported at baseline was 3 in the intervention arm and 1 in the control arm. At the third month, no visits were reported in the intervention arm and 3 were reported in the control arm. Similar numbers were observed for number of patients with hospitalizations. On complete case analysis, the Picker scale showed a between-arm difference of +18.5 [47.4] in favor of the intervention arm (Table 6).

**Table 4:** Feasibility outcomes by treatment arm.

	Intervention arm (n=24)	Control arm (n=24)
Patients from oncology practices, n	18	13
Patients from palliative care practices, n	6	11
Initiating physicians, n	10	9
Consenting initiating physicians approached who provided at least one patient, n	9	7
Patients who completed baseline and, if in the intervention arm, registered on Loop, n (%)	18 (75)	21 (87.5)
Patients recruited per initiating physician, mean (SD)	2.4 (2.2)	2.7 (2.6)
Patients with a family caregiver who participated in study, n	18	8
Teams with an additional health care provider, n	13	‒

**Table 5:** Preliminary measures of effectiveness by treatment arm, available case analysis.

		Score at each month in the intervention arm	Score at each month in the control arm	Mean observed change from baseline (SD) in the intervention arm	Mean observed change from baseline (SD) in the control arm	Unadjusted difference between change scores (SD)
**Picker Continuity and Coordination subscale, mean (SD)^a^**						
	1 month	58.7 (23.0)	64.4 (23.3)	-3.4 (29.6)	-6.7 (11.0)	3.3 (31.6)
	2 months	66.7 (27.4)	69.8 (24.7)	9.1 (25.1)	4.2 (22.8)	4.9 (33.9)
	3 months	63.5 (25.8)	60.2 (24.9)	10.2 (31.5)	-1.1 (30.3)	11.4 (43.8)
**POS, mean (SD)^b^**						
	1 month	7.5 (5.1)	8.3 (5.3)	-1.3 (4.0)	-1.0 (3.4)	-0.3 (5.2)
	2 months	7.2 (4.5)	9.2 (6.0)	0.0 (3.2)	-0.6 (4.0)	0.6 (5.1)
	3 months	8.2 (4.8)	10.3 (6.5)	0.8 (4.4)	0.5 (5.4)	0.4 (7.0)
**ESAS (Total Symptom Distress Score), mean (SD)^c^**						
	1 month	14.6 (11.8)	24.7 (15.2)	-3.0 (9.0)	1.4 (12.2)	-4.4 (15.2)
	2 months	15.2 (12.1)	21.1 (11.7)	-1.6 (9.4)	1.1 (8.0)	-2.7 (12.4)
	3 months	19.2 (9.3)	23.3 (17.0)	2.3 (10.7)	3.4 (8.7)	-1.1 (13.8)
**Patients with an emergency room visit in previous 4 weeks, n**						
	Baseline	3	1	‒	‒	‒
	1 month	1	2			
	2 months	0	2			
	3 months	0	3			
**Patients with a hospitalization in previous 4 weeks, n**						
	Baseline	3	0	‒	‒	‒
	1 month	1	1			
	2 months	0	0			
	3 months	0	3			

^a^The Picker Continuity and Coordination subscale is a proportion of total number of positive responses to total number of responses. Higher scores indicate the higher perceived continuity of care.

^b^Mean summed scores are presented for the POS with a maximum score of 40. Higher scores indicate worse quality of care.

^c^Mean summed scores are presented for the ESAS with a maximum score of 90. Higher scores indicate higher symptom distress.

**Table 6:** Complete case analysis preliminary measures of effectiveness by treatment arm.

		Score at each month in the intervention arm	Score at each month in the control arm	Mean observed change from baseline (SD) in the intervention arm	Mean observed change from baseline (SD) in the control arm	Unadjusted difference between change scores (SD)
**Picker Continuity and Coordination subscale, mean (SD)^a^**						
	Baseline	57.8 (27.5)	76.8 (21.0)	‒	‒	‒
	1 month	51.6 (20.5)	73.2 (28.3)	-6.3 (34.7)	-3.6 (11.9)	-2.7 (36.7)
	2 months	62.5 (25.0)	76.8 (24.4)	4.7 (26.7)	0.0 (21.7)	4.7 (34.4)
	3 months	65.6 (60.0)	66.1 (29.5)	7.8 (36.6)	-10.7 (30.1)	18.5 (47.4)
**POS, mean (SD)^b^**						
	Baseline	8.0 (6.0)	8.0 (6.2)	‒	‒	‒
	1 month	6.5 (3.7)	6.0 (5.9)	-1.5 (4.4)	-2.0 (3.0)	0.5 (5.3)
	2 months	8.3 (3.5)	7.1 (5.1)	0.3 (3.8)	-0.9 (2.0)	1.1 (4.3)
	3 months	8.3 (3.1)	8.4 (7.6)	0.3 (4.7)	0.4 (6.0)	-0.2 (7.6)
**ESAS (Total Symptom Distress Score), mean (SD)^c^**						
	Baseline	16.8 (10.3)	16.8 (12.8)	‒	‒	‒
	1 month	11.7 (8.0)	19.4 (13.2)	-5.1 (9.7)	2.5 (7.4)	-7.7 (12.2)
	2 months	14.3 (8.3)	20.3 (13.6)	-2.5 (11.0)	3.5 (7.4)	-6.8 (13.3)
	3 months	18.9 (8.4)	21.3 (21.4)	2.1 (11.6)	4.4 (10.3)	-2.4 (15.5)
**Patients with an emergency room visit in previous 4 weeks, n**						
	Baseline	2	1	‒	‒	‒
	1 month	1	1			
	2 months	0	0			
	3 months	0	2			
**Patients with a hospitalization in previous 4 weeks, n**						
	Baseline	2	0	‒	‒	‒
	1 month	1	0			
	2 months	0	0			
	3 months	0	3			

^a^The Picker Continuity and Coordination subscale is a proportion of total number of positive responses to total number of responses. Higher scores indicate the higher perceived continuity of care.

^b^Mean summed scores are presented for the POS with a maximum score of 40. Higher scores indicate worse quality of care.

^c^Mean summed scores are presented for the ESAS with a maximum score of 90. Higher scores indicate higher symptom distress.

## Discussion

### Principal Findings

In this pilot cRCT evaluating an online communication tool for clinical collaboration, trial feasibility conditions and implementation goals were met. The study was not powered to observe changes in outcomes between study arms, but we did observe an increase in continuity of care scores in the intervention arm at last follow-up, which was maintained on complete case analysis. Regarding the assembly of teams, though each patient identified at least one additional health care provider, only 65% of patient Loops had an additional health care provider register on the tool. Loop was primarily used for medical care management, symptom-related discussions, and appointment coordination.

### Interpretation

As a population with complex care needs [45], the advanced cancer population served as an exemplar patient population in which to evaluate Loop but also proved challenging from a participation standpoint. Although the proportion of eligible patients who consented in this study was slightly higher than two previous studies conducted at the same institution with the same population (38% here vs 10%) [36,46], a number of patients withdrew due to ill health or died over the course of the study. This was expected given the uncertainty in prognosis in this population. Instrument completion rates reflected patient retention rates, indicating that questionnaire administration was feasible despite the nature of the patient population.

Loop was designed to connect patients and caregivers to their team of health care providers in a virtual space where communication might be facilitated outside of appointments and across care settings [26]. While we did not assess differences in measures of effect for statistical significance, preliminary Picker scale results appear to support potential for this tool to improve continuity of care in future studies of adequate statistical power. Contextualized with the content of messages, the findings of this study may suggest that there were important needs that could be dealt with between appointments by using the tool, contributing to increased perceptions of continuity of care.

The care of patients with complex needs requires a redefining of the relationship between health care providers and patients to a team-based model of care that engages the patient [47]. These patients often have interdependent issues and thus require collaborative approaches to care (negotiated decision making between individuals in a synergistic manner around shared goals) over coordination between providers (alignment of functioning among independent individuals to address common needs) [48]. Here, the greater number of posts by patients over health care providers and the patient-driven content of messages (eg, Updates, Appointment Coordination) are suggestive that in this study, coordination tasks were addressed to some extent but collaboration did not occur. Given these results, we recognize that Loop in isolation did not produce collaboration, and further consideration into building relationships among these teams is required [49].

We further found that assembling the team was difficult in this study, with few health care providers from outside academic practices, who were identified by the patient, agreeing to join. Other studies have found that barriers to health care provider participation and uptake of studies of eHealth tools include lack of provider compensation and perceived worry about the burden of patient overuse [50]. Although this increased burden has not been observed thus far [51], better strategies to improve integration into clinical workflow need to be examined, especially for physicians with large patient rosters. Implementation of incentive schemes, akin to what has been done in the province of Ontario, Canada, for electronic consultations [52] may also improve uptake of eHealth tools, like Loop, into practice.

In this study, Loop was intentionally provided to teams without training. We observed that participants were able to understand and use the core functionality of Loop, that is, to post and read messages. We further observed that patients viewed their Loop more often than they posted compared to health care providers, who posted nearly as often as they viewed a Loop. This could be interpreted as showing that patients were more proactive tool users, while health care providers are more likely to wait for notifications before logging in.

### Comparison With Previous Studies

While many tools for patient-physician communication exist (frequently as secure email or part of patient portals) [19,53], few have considered the potential value of team-based communication, which is crucial for complex care scenarios or situations requiring ongoing care. At least four studies have evaluated variations of tools connecting patients and caregivers with multiple health care providers. Gulmans et al [25] found that patient groups who used their tool more often tended to have a larger care network (number of professionals registered per patient). Furthermore, Ralston et al [23], in evaluating secure messaging as part of a portal, found that messaging increased proportionally with patient morbidity, which reinforces the suggestion that messaging is of more value in complex care. While our study was too small to examine such associations, these findings support the increased value that eHealth communication tools may have as complexity of team and illness increase.

In a study by Hsiao et al [22], as has been noted elsewhere [54,55], participants felt that text-based communication may diminish the therapeutic relationship gained from in-person visits or unstructured voice-based contact (such as telephone). This suggests that such forms of communication should supplement, but not replace, appointments or calls.

The ZWIP tool, by Robben et al [24], allows for patient-provider and between-provider communication. Evaluation in frail elderly patients found that use by both patients and providers depended on provider use of the tool. Health care providers considered implementation strategies (such as training to use the tool) “very necessary” to make the most use of ZWIP. This finding may reflect the need for guided implementation to facilitate integration into clinical workflow and to improve the use of Loop.

### Limitations

The results of this study should be interpreted within the context of the study’s strengths and limitations. As a pilot study, we aimed for a sample size that was adequate to determine feasibility of implementation. However, this sample size limited the ability to test the effectiveness of the intervention. All health care providers described themselves as very comfortable with computers and worked in fully computerized practices; however, this may not be true of every medical practice, limiting study generalizability. Similarly, the complexity of clinical cases and nature of physician practice may be different elsewhere. While patients with advanced cancer are a prototypical population of patients with complex care needs and have involvement of multiple providers, similar results may not be reflected in other populations. The cRCT design, involving recruitment and randomization of initiating physicians (clusters) sequentially, and their patients prospectively, may have led to selection bias because of differential recruitment rates by provider and differences in their clinical subspecialty. We also observed that more patients died in the control arm than in the intervention arm possibly indicating unmeasured confounding. As use of the tool was voluntary, there is also a risk of confounding by indication, with patients who have more issues needing to use the tool more often, or functionally limited patients using the tool less often.

### Future Research

The results of this study will inform the next phase of research, which aims to (1) understand the conditions that affect tool adoption and assembly of teams, (2) understand the relationship between use and outcomes such as continuity and quality of care, and (3) examine the contexts and target populations where the benefits of a tool like Loop may be best realized and where the effort to assemble the care team is justified. We anticipate that by making adjustments to the implementation approach [56] through use of site champions, consideration of strategies to foster team collaboration as a co-intervention with Loop, and considering an initiating physician (the “index provider”) from those additional provider disciplines that were less represented, we may address issues with team assembly and optimize collaboration on Loop. We also expect that with a longer duration of follow-up, as could be done in other patient populations, participants’ comfort with using Loop may improve, and the content of communication may become more oriented towards care planning and decision making, over coordination.

### Conclusion

In this study, we found that it was feasible to implement Loop in clinical practice and that the tool may have the potential to improve continuity of care. We observed that Loop messages reflected message categories of medical care management, symptom reporting, and appointment coordination, among others. Usage of the tool suggests that some coordination tasks were improved but further strategies to build collaboration among team members may be needed. As an ongoing goal of eHealth development, the integration of the dynamic components of care (communication and collaboration) with the static repositories of medical records would enable a more seamless provision of health care. However challenging this may be in the current environment of multiple electronic health records across organizations, studying collaborative tools like Loop advances this goal.

## Appendix

Multimedia Appendix 1 CONSORT-EHEALTH checklist V1.6.2 [29].

Multimedia Appendix 2 Coding framework for Loop usage.

## Abbreviations

cRCT: Cluster Randomized Controlled Trial
ECOG: Eastern Cooperative Oncology Group scale
ESAS: Edmonton Symptom Assessment Scale
POS: Palliative care Outcomes Scale

## References

[1] Valderas JM, Starfield B, Sibbald B, Salisbury C, Roland M (2009). Defining comorbidity: implications for understanding health and health services. Ann Fam Med.

[2] Betancourt M, Roberts K, Bennett T, Driscoll E, Jayaraman G, Pelletier L (2011). Chronic Diseases and Injuries in Canada.

[3] Hickam D, Weiss J, Guise J, Buckley D, Motu'apuaka P, Graham E (2013). Defining Complex Care Needs.

[4] Lee SJ, Clark MA, Cox JV, Needles BM, Seigel C, Balasubramanian BA (2016). Achieving Coordinated Care for Patients With Complex Cases of Cancer: A Multiteam System Approach. J Oncol Pract.

[5] van WC, Taljaard M, Bell CM, Etchells E, Zarnke KB, Stiell IG, Forster AJ (2008). Information exchange among physicians caring for the same patient in the community. CMAJ.

[6] Smith PC, Araya-Guerra R, Bublitz C, Parnes B, Dickinson LM, Van VR, Westfall JM, Pace WD (2005). Missing clinical information during primary care visits. JAMA.

[7] Sutcliffe KM, Lewton E, Rosenthal MM (2004). Communication failures: an insidious contributor to medical mishaps. Acad Med.

[8] Haggerty JL, Reid RJ, Freeman GK, Starfield BH, Adair CE, McKendry R (2003). Continuity of care: a multidisciplinary review. BMJ.

[9] Cipriano PF, Bowles K, Dailey M, Dykes P, Lamb G, Naylor M (2013). The importance of health information technology in care coordination and transitional care. Nurs Outlook.

[10] Canada Health Infoway (2013). Opportunities for Action: A Pan-Canadian Digital Health Strategic Plan.

[11] Institute OM (2005). IOM report: patient safety--achieving a new standard for care. Acad Emerg Med.

[12] Institute of Medicine Committee on Quality of Health Care in America (2001). Crossing the quality chasm: a new health system for the 21st century.

[13] Carayon P, Hoonakker P, Cartmill R, Hassol A (2015). Using Health Information Technology (IT) in Practice Redesign: Impact of Health IT on Workflow. Patient-Reported Health Information Technology and Workflow.

[14] (2015). The World Bank.

[15] Curran V (2007). Collaborative Care.

[16] Bates DW (2015). Health Information Technology and Care Coordination: The Next Big Opportunity for Informatics?. Yearb Med Inform.

[17] Baker L, Wagner TH, Singer S, Bundorf MK (2003). Use of the Internet and e-mail for health care information: results from a national survey. JAMA.

[18] McGeady D, Kujala J, Ilvonen K (2008). The impact of patient-physician web messaging on healthcare service provision. Int J Med Inform.

[19] de Jong C, Ros WJ, Schrijvers G (2014). The effects on health behavior and health outcomes of Internet-based asynchronous communication between health providers and patients with a chronic condition: a systematic review. J Med Internet Res.

[20] Kruse CS, Bolton K, Freriks G (2015). The effect of patient portals on quality outcomes and its implications to meaningful use: a systematic review. J Med Internet Res.

[21] Walsh C, Siegler EL, Cheston E, O'Donnell H, Collins S, Stein D, Vawdrey DK, Stetson PD, Informatics Intervention Research Collaboration (I2RC) (2013). Provider-to-provider electronic communication in the era of meaningful use: a review of the evidence. J Hosp Med.

[22] Hsiao AL, Bazzy-Asaad A, Tolomeo C, Edmonds D, Belton B, Benin AL (2011). Secure web messaging in a pediatric chronic care clinic: a slow takeoff of “kids' airmail”. Pediatrics.

[23] Ralston JD, Rutter CM, Carrell D, Hecht J, Rubanowice D, Simon GE (2009). Patient use of secure electronic messaging within a shared medical record: a cross-sectional study. J Gen Intern Med.

[24] Robben SHM, Perry M, Huisjes M, van Nieuwenhuijzen L, Schers HJ, van Weel C, Rikkert MG, van Achterburg T, Heinen MM, Melis RJF (2012). Implementation of an innovative web-based conference table for community-dwelling frail older people, their informal caregivers and professionals: a process evaluation. BMC Health Serv Res.

[25] Gulmans J, Vollenbroek-Hutten M, van Germert-Pijnen L, van Harten W (2012). A web-based communication system for integrated care in cerebral palsy: experienced contribution to parent-professional communication. Int J Integr Care.

[26] Kurahashi AM, Weinstein PB, Jamieson T, Stinson JN, Cafazzo JA, Lokuge B, Morita PP, Cohen E, Rapoport A, Bezjak A, Husain A (2016). In the Loop: The Organization of Team-Based Communication in a Patient-Centered Clinical Collaboration System. JMIR Hum Factors.

[27] Schumacher KL, Stewart BJ, Archbold PG, Dodd MJ, Dibble SL (2000). Family caregiving skill: development of the concept. Res Nurs Health.

[28] Payne S, Jarrett N, Jeffs D (2000). The impact of travel on cancer patients' experiences of treatment: a literature review. Eur J Cancer Care (Engl).

[29] Eysenbach G, Consort-EHEALTH Group (2011). CONSORT-EHEALTH: improving and standardizing evaluation reports of Web-based and mobile health interventions. J Med Internet Res.

[30] Sarhill N, Walsh D, Nelson KA, LeGrand S, Davis MP (2001). Assessment of delirium in advanced cancer: the use of the bedside confusion scale. Am J Hosp Palliat Care.

[31] MacKenzie C (2002). The need for a design lexicon: Examining minimalist, performance-centered, and user-centered design. Tech Commun.

[32] Stinson JN, McGrath PJ, Hodnett ED, Feldman BM, Duffy CM, Huber AM, Tucker LB, Hetherington CR, Tse SM, Spiegel LR, Campillo S, Gill NK, White ME (2010). An internet-based self-management program with telephone support for adolescents with arthritis: a pilot randomized controlled trial. J Rheumatol.

[33] Hsieh H, Shannon SE (2005). Three approaches to qualitative content analysis. Qual Health Res.

[34] Thabane L, Ma J, Chu R, Cheng J, Ismaila A, Rios LP, Robson R, Thabane M, Giangregorio L, Goldsmith CH (2010). A tutorial on pilot studies: the what, why and how. BMC Med Res Methodol.

[35] National Research Corporation (2003). Development and Validation of the Picker Ambulatory Oncology Survey Instrument in Canada. National Research Corporation.

[36] Husain A, Barbera L, Howell D, Moineddin R, Bezjak A, Sussman J (2013). Advanced lung cancer patients' experience with continuity of care and supportive care needs. Support Care Cancer.

[37] Hughes RA, Sinha A, Aspinal F, Dunckley M, Addington-Hall J, Higginson IJ (2004). What is the potential for the use of clinical outcome measures to be computerised? Findings from a qualitative research study. Int J Health Care Qual Assur Inc Leadersh Health Serv.

[38] Pelayo-Alvarez M, Perez-Hoyos S, Agra-Varela Y (2013). Reliability and concurrent validity of the Palliative Outcome Scale, the Rotterdam Symptom Checklist, and the Brief Pain Inventory. J Palliat Med.

[39] Bajwah S, Ross J, Wells A, Mohammed K, Oyebode C, Birring S, Patel AS, Koffman J, Higginson IJ, Riley J (2015). Palliative care for patients with advanced fibrotic lung disease: a randomised controlled phase II and feasibility trial of a community case conference intervention. Thorax.

[40] Bruera E, Kuehn N, Miller MJ, Selmser P, Macmillan K (1991). The Edmonton Symptom Assessment System (ESAS): a simple method for the assessment of palliative care patients. J Palliat Care.

[41] Guerriere DN, Ungar WJ, Corey M, Croxford R, Tranmer JE, Tullis E, Coyte PC (2006). Evaluation of the ambulatory and home care record: Agreement between self-reports and administrative data. Int J Technol Assess Health Care.

[42] Harris PA, Taylor R, Thielke R, Payne J, Gonzalez N, Conde JG (2009). Research electronic data capture (REDCap)--a metadata-driven methodology and workflow process for providing translational research informatics support. J Biomed Inform.

[43] Lancaster GA, Dodd S, Williamson PR (2004). Design and analysis of pilot studies: recommendations for good practice. J Eval Clin Pract.

[44] Charlson ME, Pompei P, Ales KL, MacKenzie CR (1987). A new method of classifying prognostic comorbidity in longitudinal studies: development and validation. J Chronic Dis.

[45] Armes J, Crowe M, Colbourne L, Morgan H, Murrells T, Oakley C, Palmer N, Ream E, Young A, Richardson A (2009). Patients' supportive care needs beyond the end of cancer treatment: a prospective, longitudinal survey. J Clin Oncol.

[46] Zimmermann C, Swami N, Krzyzanowska M, Hannon B, Leighl N, Oza A, Moore M, Rydall A, Rodin G, Tannock I, Donner A, Lo C (2014). Early palliative care for patients with advanced cancer: a cluster-randomised controlled trial. Lancet.

[47] Weiner SJ, Barnet B, Cheng TL, Daaleman TP (2005). Processes for effective communication in primary care. Ann Intern Med.

[48] Keast R, Brown K, Mandell M (2007). Getting The Right Mix: Unpacking Integration Meanings and Strategies. International Public Management Journal.

[49] Kuziemsky CE, O'Sullivan TL (2015). A model for common ground development to support collaborative health communities. Soc Sci Med.

[50] Crotty BH, Tamrat Y, Mostaghimi A, Safran C, Landon BE (2014). Patient-to-physician messaging: volume nearly tripled as more patients joined system, but per capita rate plateaued. Health Aff (Millwood).

[51] Sullivan M (2008). Dermatology News.

[52] Ontario Medical Association (2015). Ontario Health Insurance Plan Payments for E-Consultation Services for Referring Physicians (K738). OHIP Schedule of Benefits 2015.

[53] Wildevuur SE, Simonse LW (2015). Information and communication technology-enabled person-centered care for the “big five” chronic conditions: scoping review. J Med Internet Res.

[54] Katz SJ, Moyer CA, Cox DT, Stern DT (2003). Effect of a triage-based E-mail system on clinic resource use and patient and physician satisfaction in primary care: a randomized controlled trial. J Gen Intern Med.

[55] Cartwright M, Hirani SP, Rixon L, Beynon M, Doll H, Bower P, Bardsley M, Steventon A, Knapp M, Henderson C, Rogers A, Sanders C, Fitzpatrick R, Barlow J, Newman SP, Whole Systems Demonstrator Evaluation Team (2013). Effect of telehealth on quality of life and psychological outcomes over 12 months (Whole Systems Demonstrator telehealth questionnaire study): nested study of patient reported outcomes in a pragmatic, cluster randomised controlled trial. BMJ.

[56] Damschroder LJ, Aron DC, Keith RE, Kirsh SR, Alexander JA, Lowery JC (2009). Fostering implementation of health services research findings into practice: a consolidated framework for advancing implementation science. Implement Sci.

