# Supplementary material for: My Team of Care Study: A Pilot Randomized Controlled Trial of a Web-Based Communication Tool for Collaborative Care in Patients With Advanced Cancer
Source: J Med Internet Res. 2017 Jul 18;19(7):e219. doi: 10.2196/jmir.7421 (PMC5539387; doi:10.2196/jmir.7421)
Supplement: Multimedia Appendix 2 [file jmir_v19i7e219_app2.pdf]

Multimedia Appendix 2
Coding Framework for Loop Usage
Method of coding categories: The message exchange on each Loop was independently reviewed by two coders (TV and AB) for text that reflected any of the categories described below, at least once. Categories were assigned to messages and any responses or follow-up posts. Categories were not quantified but assigned only once per Loop because of the challenge in defining the beginning and conclusion of an exchange. If multiple categories were perceived in a single post, then each was included as identified in the Loop. Introductions posted by administrators were not counted.

<u>Category definitions and sample quotations reflecting the label</u>
Abbreviations
Pt- patient
HCP- healthcare provider

1. Introductions- messages that introduce team participants on the tool
    1) "Got it! Welcome to Loop [pt]! I'm glad to see that you and [HCP] have a handle of the Attention To function; in this way, you can direct messages to individuals which will alert them through their email. There is also a Tag Issue feature (in the compose message box), where you can label conversations around a particular issue, for example, a symptom. Messages can be filtered (on the left side of the screen) as well."

2. Symptoms- messages that relate new or updates to symptoms
    1) "I've had an increase in hoarseness and swallowing difficulties as well as episodes of tetany to my left hand mostly affect [HCP]. I feel the [test 1] and [test 2] are needed especially due to the recent changes in symptoms, location of my cancer and its metastatic routes."

3. Appointment coordination- messages related to scheduling and booking appointments.
    1) "Dear Dr [HCP1] and Dr [HCP2] Sorry if you receive this twice but I think my previous message got deleted. My next appointment with you is [date]."

    2) "Dr. [HCP1]; Sorry to inform you that I messed up the schedule for my CT Scan and your appointment. I missed taking the premedication this morning and have a rescheduled scan on the [date 1]. The earliest appointment the clinic could reschedule with you is [date 2]."

4. Administrative/tool set-up- related to assembling Loop team members, technical issues and usage instructions
    1) "Hi [pt], yes as of right now those are the team members who have activated their loops. Dr. [HCP1] and Dr. [HCP2] invitations are pending. Best, [study administrator]"

5. Medication information/changes- related to providing information (but not prescription or administration of medication) of medication, dosage etc.
   1) “Hi [pt], I hope that you are doing well since our last meeting. Remember the medication ritalin (other name - methylphenidate) is there if you need it for fatigue. Feel free to post any issues or concerns. talk soon! [HCP]”

6. Prescription renewal- messages related to renewal of medication
   1) “I would really appreciate it if you can send a prescription (prefrebly for two months)for [drug] to the pharmacy. I have only x pills left. Fax: 12345789 Thank you [pt]”

7. Prescription of new medication- messages related to new prescription of medications
   1) From patient: "Hello, I was wondering if you can kindly send a prescription for Imodium to the pharmacy. I want to give it a new shot and see if I can get reimbersment from my insurance co. Thank you"

      From HCP: “Hi [pt] That's fine, I've sent a script for 280 tablets (and one repeat) for you to try. The generic name is Loperamide, it's exactly the same as Immodium, which is one of the brand names. With thanks [HCP]"

8. Medical care updates- messages related to updating either the physician or the patient on status
   1) From patient: “Sugars for the first 15 days were from 7 - 13 with many double digit counts. Things have settled down some so that since the first of July the range has been 4.8 to 9.1 with none of the low counts I was experiencing before the embolization.”

9. Medical care management- related to direction, management and provision of care, including ordering tests and administration of medication.
   1) “Hi [HCP1] and [HCP2], Dr. [HCP3] said you need a nurse to administer an injection next month. Do you know which MD I need to call to get the order? Thanks,”

   2) “Good Morning I left on a message with Dr [HCP’s] office regarding this issue but I figured this would also be a good idea. I returned from [location 1] on Friday and went through 6 weeks of mail. I found my [hospital name] appointment letter and I'm concerned that the CTs that are ordered are chest abdo and pelvis. Can you please add head and neck? …I feel the head and neck are needed especially due to the recent changes in symptoms, location of my cancer and its metastic routes. Also, the bloodwork does not have ECG added to it and this presents me with difficulty with the ECG tech as I have to talk her/him into doing the ECG without an online order. Thank you for your time.”

Appendix Table 1 Responses to computer usage questionnaires by user group

	Intervention arm	Control arm
**Patients**	**(n=21)**	**(n=21)**
Computer usage – no. (%)		
At work	6 (28.6)	6 (28.6)
At home	17 (81.0)	19 (90.5)
Internet access	17 (81.0)	19 (90.5)
Any use of other devices – no. (%)		
Tablet	7 (33.3)	12 (57.1)
Smartphone	14 (66.7)	14 (66.7)
None	2 (9.5)	1 (4.8)
Daily use of computers or related devices – no. (%)		
Not at all	4 (19.1)	1 (4.8)
≤2 hr	6 (28.6)	13 (61.9)
3-7 hrs	8 (38.1)	6 (28.6)
>7 hrs	3 (14.3)	1 (4.8)
Daily internet use – no. (%)		
Not at all	4 (19.1)	1 (4.8)
≤2 hr	10 (47.6)	13 (61.9)
3-7 hrs	7 (33.3)	6 (28.6)
>7 hrs	0 (0)	1 (4.8)
Comfortable using:		
Computers (median, IQR 1 to 4 (most))	4.0 (2.0)	3.0 (2.0)
Smartphone or tablet (median, IQR 1 to 4 (most))	2.0 (3.0)	3.0 (2.0)
Internet (median, IQR 1 to 4 (most))	4.0 (2.0)	3.0 (2.0)
Email (median, IQR 1 to 4 (most))	3.0 (3.0)	4.0 (1.0)
Instant messaging (median, IQR 1 to 4 (most))	2.0 (3.0)	3.0 (3.0)
Social media (median, IQR 1 to 4 (most))	1.0 (2.0)	3.0 (4.0)
**Initiating Physicians**	**(n=10)**	**(n=9)**
Computer usage – no. (%)		
At work	10 (100)	9 (100)
At home	10 (100)	9 (100)
Internet access	10 (100)	9 (100)
Any use of other devices – no. (%)		
Tablet	1 (10)	1 (11.1)
Smartphone	9 (90)	7 (77.8)
Daily use of computers or related devices – no. (%)		
Not at all	0 (0)	0 (0)
≤2 hr	0 (0)	0 (0)
3-7 hrs	6 (60)	8 (88.9)
>7 hrs	4 (40)	1 (11.1)

Daily internet use – no. (%)		
Not at all<br>≤2 hr<br>3-7 hrs<br>>7 hrs	0 (0)<br>1 (10)<br>8 (80)<br>1 (10)	0 (0)<br>4 (44.4)<br>5 (55.6)<br>0 (0)
Comfort with:		
Computers (median, IQR 1 to 4 (most))<br>Smartphone or tablet (median, IQR 1 to 4 (most))<br>Internet (median, IQR 1 to 4 (most))<br>Email (median, IQR 1 to 4 (most))<br>Instant messaging (median, IQR 1 to 4 (most))<br>Social media (median, IQR 1 to 4 (most))	3.5 (1.0)<br>3.0 (1.0)<br>4.0 (1.0)<br>4.0 (0.0)<br>3.0 (2.0)<br>0.5 (3.0)	4.0 (0.0)<br>4.0 (1.0)<br>4.0 (0.0)<br>4.0 (0.0)<br>4.0 (1.0)<br>2.0 (1.0)
**Caregivers of consented patient participants**	**(n=18)**	**(n=8)**
Computer usage – no. (%)		
At work<br>At home<br>Internet access	10 (55.6)<br>15 (83.3)<br>15 (83.3)	4 (50.0)<br>7 (87.5)<br>7 (87.5)
Any use of other devices – no. (%)		
Tablet<br>Smartphone	11 (61.1)<br>10 (55.6)	3 (37.5)<br>4 (50.0)
Daily use of computers or related devices – no. (%)		
Not at all<br>≤2 hr<br>3-7 hrs<br>>7 hrs	0 (0)<br>7 (38.9)<br>7 (38.9)<br>1 (5.6)	0 (0)<br>5 (62.5)<br>2 (25.0)<br>0 (0)
Daily internet use – no. (%)		
Not at all<br>≤2 hr<br>3-7 hrs<br>>7 hrs	0 (0)<br>9 (50.0)<br>5 (27.8)<br>1 (5.6)	0 (0)<br>6 (75.0)<br>1 (12.5)<br>0 (0)
Comfort with:		
Computers (median, IQR 1 to 4 (most))<br>Smartphone or tablet (median, IQR 1 to 4 (most))<br>Internet (median, IQR 1 to 4 (most))<br>Email (median, IQR 1 to 4 (most))<br>Instant messaging (median, IQR 1 to 4 (most))<br>Social media (median, IQR 1 to 4 (most))	3.0 (1.0)<br>3.0 (2.0)<br>3.0 (1.0)<br>4.0 (1.0)<br>3.0 (1.0)<br>2.0 (3.0)	3.0 (2.0)<br>2.0 (2.0)<br>4.0 (2.0)<br>4.0 (1.0)<br>2.0 (2.0)<br>1.0 (3.0)

IQR= interquartile range
